# The efficacy and safety of azithromycin in asthma: A systematic review

**DOI:** 10.1111/jcmm.13919

**Published:** 2019-01-19

**Authors:** Bao‐Ping Tian, Nanxia Xuan, Yesong Wang, Gensheng Zhang, Wei Cui

**Affiliations:** ^1^ Department of Critical Care Medicine The Second Affiliated Hospital Zhejiang University School of Medicine Hangzhou Zhejiang China

**Keywords:** adverse events, asthma, azithromycin, life quality, lung function, systematic review

## Abstract

Azithromycin is a potential therapeutic choice for asthma control, which is a heterogeneous airway inflammatory disease. Because of variable findings, we intend to evaluate the therapeutic effect and safety of azithromycin in asthma. Databases, including PubMed, EMBASE, Cochrane, and CNKI until 31 December 2017, were searched to identify available randomised controlled trials regarding azithromycin treatment for asthma. We identified seven studies involving 1520 cases that met our criteria. The mean difference for lung function (FEV_1_, FVC, PEF), symptom assessment (ACQ, AQLQ), airway inflammation, and risk ratios for adverse events were extracted. Chi‐square and *I* ^2^ tests were applied to evaluate the heterogeneity among the studies towards each index with a random effect model or a fixed effect model. Pooled analysis shows that azithromycin administration results in no significant improvement in FEV_1_ (MD: 0.09, 95% CI −0.10 to 0.29, *P* = 0.36), PEF (MD: 11.76; 95% CI, −2.86 to 26.38, *P* = 0.11), total airway inflammatory cells (MD: −0.29; 95% CI, −1.38 to 0.80, *P* =  0.60), ACQ (MD: 0.05; 95% CI, −0.08 to 0.19, *P* = 0.44), and AQLQ (MD: 0.12; 95% CI, −0.02 to 0.26, *P* =  0.10). Moreover, no significant difference was detected in adverse events (Risk ratio 0.99; 95% CI, 0.82‐1.19, *P* = 0.90). These findings demonstrate no beneficial clinical outcome of azithromycin in asthma control, and we propose that further prospective cohorts are warranted.

## 1 INTRODUCTION

Bronchial asthma is a heterogeneous disease that is characterised by airway inflammation, mucus over‐secretion, airway hyper‐responsiveness, and eventually, airway wall remodelling.^1^ Asthma affects 5%‐16% and up to 334 million people worldwide and result in substantial medical expenditures.^2^, ^3^ Currently, daily inhaled corticosteroids combined with long‐acting β2 agonists are the first‐line strategy of asthma treatment. Other drugs, including leukotriene‐receptor antagonists, theophylline, long‐acting anti‐cholinergics, and even oral corticosteroids, are added for those asthma patients who were not well controlled.^4^ Besides antibacterial effects, macrolides such as azithromycin are also reported to have immunomodulatory and anti‐inflammatory effects in airway inflammatory disease, including cystic fibrosis, bronchiectasis, exacerbation of chronic obstructive pulmonary disease, and severe asthma.^5^, ^6^, ^7^, ^8^ Although some clinical trials have tested the therapeutic effect of azithromycin in asthma, the conclusions were inconsistent. Here, we present the results of a systematic review aimed to provide a summary of the efficacy and safety of administering azithromycin in asthma patients.

## 2 METHODS

### 2.1 Data sources and search strategy

All of the literature reporting the effect of azithromycin in patients with asthma was systematically retrieved through the databases, including PubMed, EMBASE, and Cochrane Controlled Trials Register databases until 31 December 2017. The search keywords (“Azithromycin” AND “Asthma” OR “Bronchial asthma” OR “Allergic airway inflammation”) were used to extract the related articles, without language restriction. The China National Knowledge Internet (CKNI) database was also searched from inception to December 2017 using equivalent Chinese terms. To identify other potentially eligible articles, studies were further searched manually by reviewing titles, abstracts, and full texts using EndNote X8 software. A manual search was also conducted on primary studies and review articles, and manufacturers’ websites for trial information to avoid missing potential articles. This process was performed independently by two researchers.

### 2.2 Inclusion and exclusion criteria

The inclusion criteria for considering studies of this systematic review were as follows: (a) all of the participants were definitely diagnosed as asthma; (b) studies were designed as randomised controlled trials; (c) azithromycin as the intervention treatment compared with placebo or azithromycin in combination with other therapies compared with other therapies alone; and (d) outcome reported lung function, airway inflammation, exacerbations, symptom control or adverse events. Studies were excluded if they had any of the following characteristics: (a) text without data about participant characteristics or outcome, such as guidelines, reviews, comments, correspondences, editorials, case reports; (b) studies were not performed in humans, or conducted in ex vivo cells or animals; (c) studies only analysed participants with a special occupation (eg, athletes and farmers). The eligible articles were judged and selected by two researchers independently.

### 2.3 Data extraction

Two investigators independently abstracted the following information in eligible articles: study design, general characteristics of patients (sample size, age, number of female or male in each trial, country/area or gender), baseline of asthma severity, dosages and therapeutic process of azithromycin, the administration on asthma patients in placebo groups, duration of follow‐up, and the primary and secondary outcome (forced vital capacity, FVC; forced expiratory volume in 1 second, FEV_1_; peak expiratory flow, PEF; percentage of sputum eosinophils/neutrophils; asthma exacerbation rate; Asthma Quality of Life Questionnaire, AQLQ; Asthma Control Questionnaire, ACQ; and adverse events). Some data were calculated with available data by Review Manager (RevMan, version 5.3.0., Cochrane Collaboration, Oxford, UK), if they were not provided directly in texts. Any discrepancies were resolved by the third reviewer after assessing the original articles.

### 2.4 Study quality assessment

The independent quality assessments for each of the RCTs were conducted according to the *Cochrane Handbook for Systematic Reviews of Interventions* by two authors.^9^ A total of seven items (random sequence generation, allocation concealment, blinding of participants and personnel, blinding of outcome assessment, incomplete outcome data, selective reporting, and other bias) were applied for evaluating the risk of bias. The potential bias was graded as high, low, or unclear risk. Any divergence was settled by discussion with the third investigator.

### 2.5 Statistical analysis

The changes in lung function (FEV_1_, FVC, PEF), symptom assessment (ACQ, AQLQ), airway inflammation, and adverse events rates were analysed in this systematic review. Chi‐square and *I* ^2^ tests were used to evaluate the heterogeneity among the studies towards each index. *P* (χ^2^ test) < 0.10 or *I* ^2^ ≥ 50% were considered statistical heterogeneity. A random effect model or fixed effect model was applied for meta‐analysis with (*P* < 0.10, *I* ^2^ ≥ 50%) or without heterogeneity (*P* > 0.1, *I* ^2^ < 50%), respectively. The comparison of the outcome between the azithromycin and placebo was conducted using Review Manager 5.3 (Revman, The Cochrane Collaboration, Oxford, UK). *P*‐values < 0.05 were considered statistically significant. Kappa index was used to assess the consistency between the authors that performed this review; all values of that index were ≥ 0.75, indicating an acceptable consistency.

## 3 RESULTS

### 3.1 Characteristics of included studies

**Figure 1:**
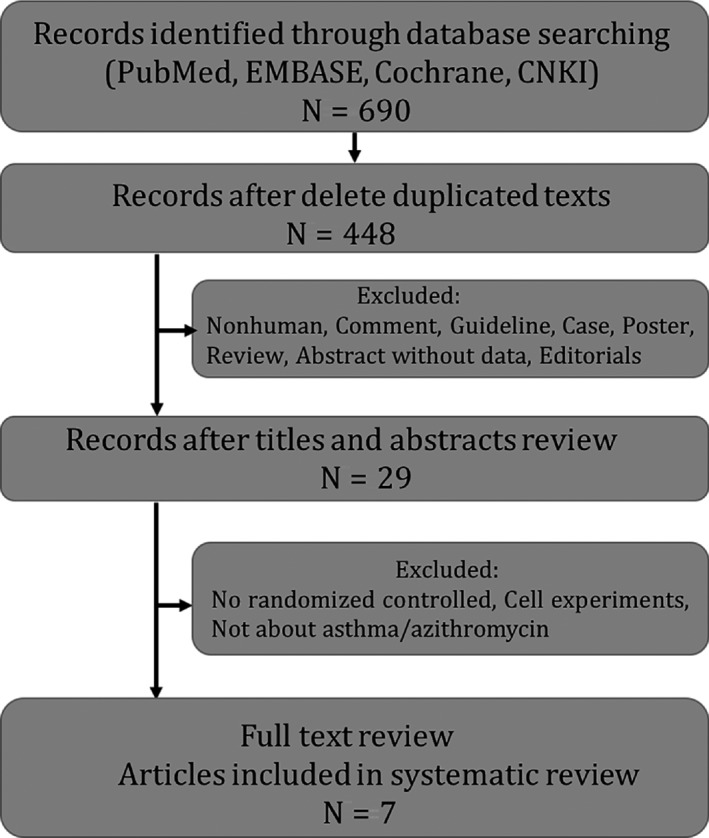
Flow diagram for the literature search

Six‐hundred‐ninety studies were selected by searching PubMed, EMBASE, the Cochrane database, and CNKI, and 448 studies remained after removing duplicated texts. From the titles and abstracts, 419 studies were excluded because they were identified as guidelines, posters, comments, editorials or reviews, or involving ex vivo experiments, animal models or any other of the exclusion criteria. Twenty‐nine studies were selected for the further full‐text review, and finally, as shown in Figure 1, a total of seven studies with 1520 participants were selected for the systematic review and quantitative analysis.^10^, ^11^, ^12^, ^13^, ^14^, ^15^, ^16^ All of the included studies were designed as randomised, double‐blind, placebo‐controlled clinical trials. Only one study was conducted in children, and the remaining six studies were conducted in adults (≥18‐years‐old). Johnston et al conducted a study in patients requesting emergency care for acute asthma exacerbations,^15^ while the six other studies analysed the effects of azithromycin on stable or persistent asthma. All of the asthma patients in the placebo groups in each study received ICS plus LABA as the usual care for asthma control; the ICS used in these studies included fluticasone or beclomethasone dipropionate. However, Hahn DL, Johnston SL, and Gibson PG have not provided details of the treatment in the placebo groups.^10^, ^15^, ^16^ The characteristics of the studies are shown in Table 1.

**Table 1:** Characteristics and designs of the included studies

Study	Study design	Female/Patients	Mean age (y)	Inclusion criteria	Baseline treatment	Azithromycin intervention	Follow‐up	Primary and secondary outcomes
Hahn^10^	Multisite, Randomised, allocation‐concealed, blinded, placebo‐controlled parallel	23/45	47.67	Age ≥ 18 y, persistent, stable and present for more than 3 mo prior to enrolment, eligible patients remained in the same severity class and had no acute exacerbations	Usual care for asthma from their primary physician	600 mg/d for 3 consecutive days, followed by 600 mg/wk for an additional 5 wk	3 mo	AQLQ, asthma symptoms, rescue medication use
Piacentini^11^	Randomised, double‐blind, placebo‐controlled	4/16	13.37	Asthmatic children with no clinical sign or symptom of airway infection at the time of the study	Long‐term low dose ICS: either fluticasone 100‐200 g/day, or beclomethasone dipropionate 200–400 g/day	10 mg/kg body weight/day for three consecutive days every week	8 wk	Lung function, bronchial hyper‐responsiveness, airway inflammation
Hahn^12^	Randomised, double‐blind, placebo‐controlled, effectiveness	51/75	46.54	Age ≥ 18 y, persistent asthma ≥ 6 mo before enrolment, symptomatic ≥ 2 d per week and/or ≥2 nights per month or in exacerbation	ICS + LABA and/or Leukotriene inhibitor, and/or oral prednisone	600 mg/d for 3 d followed by 600 mg weekly for 11 wk	48 wk	Asthma symptom scores, AQLQ, ACQ, exacerbations, other respiratory illnesses, off‐study antibiotic use, adverse events, asthma‐controller medications use, self‐reported asthma improvement
Cameron^14^	Randomised, double‐blind, parallel	40/77	44.62	Age 18‐70 y, current smokers (≥ 5 packs‐y history), chronic asthma >1‐y duration, free of exacerbation and respiratory tract infection for a minimum 6‐wk period prior to randomisation	ICS equivalent to 400 mg beclometasone + LABA ≥ 4 wk	250 mg/d	12 wk	PEF, PC20, FEV_1_, FeNO50, ACQ, LCQ, AQLQ
Brusselle^13^	Multicentre, randomised, double‐blind, placebo‐controlled parallel	67/109	53.00	Age 18‐75 y, persistent asthma, GINA step 4 or 5, Receive high doses of ICS (≥1000 mg fluticasone or equivalent) + LABA ≥6 months, at least two independent severe asthma exacerbations requiring systemic corticosteroids and/or LRTI requiring antibiotics within the previous 12 mo, FeNO level below the upper limit of normal, never‐smokers or ex‐smokers with a smoking history of ≤10 pack‐year	High doses ICS (≥1000 mg fluticasone or equivalent) + LABA ≥6 mo	250 mg/d for 5 days and then 250 mg three times a week	26 wk	Asthma exacerbations, and/or LRTI requiring antibiotics, FEV_1_, PEF, AQLQ, ACQ, adverse events, serious adverse events and adverse events leading to discontinuation
Johnston^15^	Multicentre, randomised, double‐blind, placebo‐controlled	139/199	37.61	Age 18‐55 y with any smoking history, age 56‐65 y with < 20 pack‐year smoking history, or older than 65 with <5 pack‐year smoking history. Asthma ≥6 mo, exacerbation symptom score severity 4.16, PEF 69.4% of predicted, FEV_1_, 64.8% of predicted, FEV_1_/FVC 69.2%	Not mentioned	500 mg, on day 1, 5 and 10	10 d	Diary card summary symptom score, AQLQ, PEF, FEV_1_, FVC, time to 50% reduction in symptom score
Gibson^16^	Multicentre, randomised, double‐blind, placebo‐controlled parallel	255/420	60.52	Age ≥ 18 y, variable airflow obstruction from bronchodilator response, airway hyper‐responsiveness, or increased peak flow variability, and were currently symptomatic with at least partial loss of asthma control (ACQ6 ≥ 0.75)	Not mentioned	500 mg, three times weekly	48 wk	Asthma exacerbations, AQLQ, ACQ, lung function, induced sputum cell counts, antibiotic courses, microbial assessments, adverse events

### 3.2 Risk of bias for included studies

We determined that six out of the seven studies were at low risk in both of random sequence generation and allocation concealment. One study had not provided details for selection bias and was assessed as unclear risk.^14^ Three studies stated that all of participants and investigators remained masked during the study, and were considered to be at low risk,^10^, ^12^, ^16^ and no detailed information for performance bias and detection bias was provided in the other four studies. We considered all of the seven studies contributing data to be at low risk in attrition bias and reporting bias (Table 2).

**Table 2:** Risk of bias of the included studies

Study	Random sequence generation (selection bias)	Allocation concealment (selection bias)	Blinding of participants/personal (performance bias)	Blinding of outcome assessment (detection bias)	Incomplete outcome data (attrition bias)	Selective reporting (reporting bias)
Hahn^10^	Low risk	Low risk	Low risk	Low risk	Low risk	Low risk
Piacentini^11^	Low risk	Low risk	Unclear risk	Unclear risk	Low risk	Low risk
Hahn^12^	Low risk	Low risk	Low risk	Low risk	Low risk	Low risk
Cameron^14^	Unclear risk	Unclear risk	Unclear risk	Unclear risk	Low risk	Low risk
Brusselle^13^	Low risk	Low risk	Unclear risk	Unclear risk	Low risk	Low risk
Johnston^15^	Low risk	Low risk	Unclear risk	Unclear risk	Low risk	Low risk
Gibson^16^	Low risk	Low risk	Low risk	Low risk	Low risk	Low risk

### 3.3 Lung function changes

The seven included articles mentioned lung function changes after azithromycin or placebo treatment (Table S1). FEV_1_ and PEF were analysed as follows.

#### 3.3.1 FEV_1_

**Figure 2:**
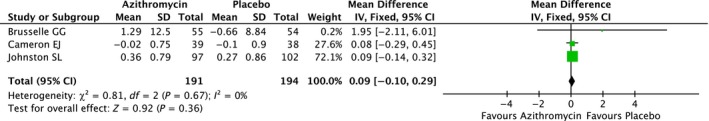
Forest plot estimating the difference in FEV_1_ changes between azithromycin and placebo treatment in asthma

Comprehensive analysis based on three studies^13^, ^14^, ^15^ indicated no significant improvement of FEV_1_ in 191 patients treated with azithromycin compared 194 patients treated with placebo (MD: 0.09, 95% CI −0.10 to 0.29, *P* = 0.36). Statistical heterogeneity was not observed among the studies (*I* ^2^ = 0%, *P* = 0.67) (Figure 2). In addition to FEV_1_, Johnston SL also found no significant difference in change compared to placebo in any measures of lung function in FVC (mean difference, −0.038; 95% CI, −0.166 to 0.243), FEV_1_/FVC (mean difference, 1.379; 95% CI, −1.559 to 4.316).^15^ In Piacentini GL's study,^11^ no significant change was observed in lung function before and after treatment. Moreover, these authors also evaluated the bronchial hyper‐responsiveness (BHR) expressed as the dose‐response slope (DRS) of FEV_1_ falls after hypertonic saline inhalation. DRS (percent fall of FEV_1_/mL) decreased from 2.75 ± 2.12 to 1.42 ± 1.54 (mean ± SD) in azithromycin‐treated children (*P* = 0.02), and decreased (non‐significantly) from 1.48 ± 1.75 at baseline to 1.01 ± 1.38 at the endpoint of the study in the placebo group (*P* = 0.21). As one of the secondary outcome, lung function changes were also assessed by Gibson et al,^16^ who found no obvious improvement in FEV_1_ after azithromycin administration compared with placebo.

#### 3.3.2 PEF variability

**Figure 3:**
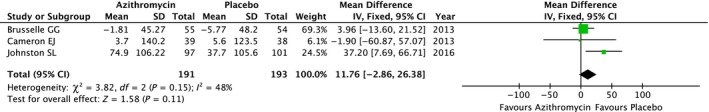
Forest plot estimating the difference in PEF changes between azithromycin and placebo treatment on asthma

Three studies^13^, ^14^, ^15^ evaluated PEF changes from baseline in adults. The pooled analysis revealed no significant PEF improvement in azithromycin compared with the placebo group (MD: 11.76; 95% CI, −2.86 to 26.38, *P* = 0.11), in a fixed effect model (*I* ^2^ = 48%, *P* = 0.15) (Figure 3). Brusselle GG reported no significant difference between the groups in the change from baseline of FEF (mean difference, 3.96; 95% CI, −15.40 to 23.32; *P* = 0.686)^13^; and there was also no statistically significant PEF improvement in azithromycin‐treated asthma patients in the studies by Cameron EJ (mean difference, −10.3; 95% CI, −47.1 to 26.4; *P* = 0.58),^14^ and Johnston (mean difference, 19.57; 95% CI, −6.81 to 45.94).^15^ We performed leave‐1‐out analyses to explore the sources of heterogeneity. By excluding the study of Johnston et al, heterogeneity was reduced to 0% (*I* ^2^ = 0%, *P* = 0.85) and with no significant difference in PEF (MD: 3.48; 95% CI, −13.35 to 20.31, *P* = 0.69), which further confirmed that the heterogeneity was mainly driven by the Johnston SL study.

### 3.4 Airway inflammation

**Figure 4:**
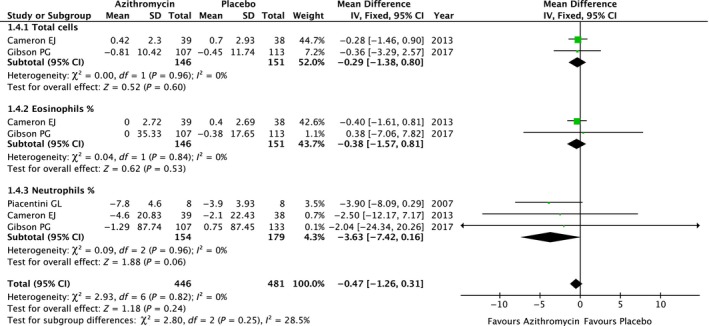
Comparison of azithromycin vs placebo in airway total inflammatory cells counts, eosinophil, and neutrophil percentage

Airway inflammation is the distinct characteristic of asthma pathology. Three studies^11^, ^14^, ^16^ reported inflammatory cells changes in sputum after azithromycin treatment. The meta‐analysis revealed that the total inflammatory cells (MD: −0.29; 95% CI, −1.38 to 0.80, *P* = 0.60),^14^, ^16^ eosinophil percentage (MD: −0.38; 95% CI, −1.57 to 0.81, *P* = 0.53),^14^, ^16^ and neutrophil percentage (MD: −3.63; 95% CI, −7.42 to 0.16, *P* = 0.06)^11^, ^14^, ^16^ in sputum were not significantly reduced in the azithromycin condition compared to placebo. Totally, the overall effect of three studies with 927 participants revealed that azithromycin on sputum inflammatory cell changes was not statistically significant (MD: −0.47; 95% CI, −1.26 to 0.31, *P* = 0.25) (Figure 4).

### 3.5 Asthma Control Questionnaire (ACQ) score

**Figure 5:**
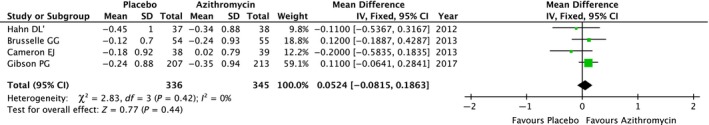
The effect of azithromycin vs placebo on ACQ

Four studies^12^, ^13^, ^14^, ^16^ with 681 asthma patients contributed asthma control measured by ACQ, indicating no statistically significant effect in favour of azithromycin vs placebo (MD: 0.05; 95% CI, −0.08 to 0.19, *P* = 0.44) (Figure 5).

### 3.6 Asthma Control and Quality of Life Assessment (AQLQ) score

**Figure 6:**
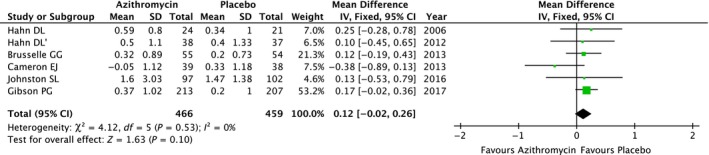
The effect of azithromycin vs placebo on AQLQ

In comparing the group receiving azithromycin to placebo, there was no difference in improvement of AQLQ based on meta‐analysis of six studies,^10^, ^12^, ^13^, ^14^, ^15^, ^16^ (MD: 0.12; 95% CI, −0.02 to 0.26, *P* = 0.10) (Figure 6). The pooled results did not change after sensitivity analysis with the removal of one study. As Brusselle GG reported, there was a significant improvement in the AQLQ score in the azithromycin group (0.32 ± 0.89; 95% CI, 0.08‐0.57, *P* = 0.011) compared with a non‐significant trend in the placebo group. Nevertheless, no significant differences between two groups in the change from baseline in AQLQ score were observed (mean difference 0.12; 95% CI, −0.20 to 0.44; *P* = 0.467). Although Hahn et al indicated no obvious improved AQLQ score from baseline after azithromycin treatment, they detected ameliorated overall asthma symptoms (including cough, wheeze, shortness of breath, and sleep disturbance due to asthma) in the azithromycin group (+0.55) and worsened symptoms in the placebo‐treated group (−0.13), suggesting a statistically significant difference (*P* = 0.04).

### 3.7 Asthma exacerbation

Three articles in the seven included studies describing the severe exacerbations of asthma,^12^, ^13^, ^16^ Both Hahn and Brusselle GG reported no significant differences between the experimental and control groups in exacerbation frequency. For the reasons of the heterogeneous phenotype of asthma, Brusselle GG also assessed the severe exacerbation of multiple airway inflammatory types, such as eosinophilic asthma and non‐eosinophilic asthma (blood eosinophilia ≤ 200/ml). For severe eosinophilic asthma, the severe exacerbation rate was significantly higher in the azithromycin group than in placebo group (Estimated rate ratio 2.19; 95% CI, 1.01‐4.73, *P* = 0.046). Interestingly, in patients with non‐eosinophilic severe asthma, there was a trend towards a decreased rate of severe exacerbation after azithromycin treatment (estimated rate ratio 0.42; 95% CI, 0.17‐1.00, *P* = 0.050). In the AMAZES trial,^16^ Gibson analysed the incidences of asthma exacerbation in the subgroups of eosinophilic asthma (sputum eosinophils ≥3% or blood eosinophil count ≥ 300/μL) and non‐eosinophilic asthma (sputum eosinophils <3% or blood eosinophil count < 300/μL). Overall, there was a significant reduction in the incidence of total asthma exacerbations, including moderate and severe in azithromycin group (incidence rate ratio 0.59; 95% CI, 0.47‐0.74, *P* < 0.0001). Moreover, the number of patients who experienced at least one asthma exacerbation was lower in the azithromycin group (94, 44%) than in the placebo group (127, 61%) (*P* < 0.0001). Subgroup analysis showed azithromycin reduced asthma exacerbations in both eosinophilic asthma (incidence rate ratio 0.52, 95% CI 0.29‐0.94; *P* = 0.030) and non‐eosinophilic asthma (incidence rate ratio 0.66; 95% CI, 0.47‐0.93; *P* = 0.019). The detailed information on asthma exacerbations is shown in Table S2.

### 3.8 Adverse events

**Figure 7:**
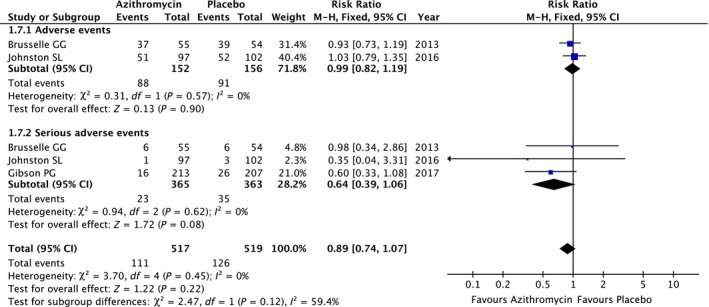
The difference in treat‐related adverse events level between azithromycin and placebo

Two cohorts presented the comparison between azithromycin and placebo treatment in total adverse events.^13^, ^15^ A pooled analysis applied in a fixed effects model (heterogeneity *I* ^2^ = 0%, *P* = 0.57) revealed no significant difference (Risk ratio 0.99; 95% CI, 0.82‐1.19; *P* = 0.90) (Figure 7). Similarly, a meta‐analysis result based on the three studies with 728 asthma patients,^13^, ^15^, ^16^ indicated no significant between‐group differences in serious adverse events (Risk ratio 0.64; 95% CI, 0.39‐1.06; *P* = 0.08) (Figure 7). Overall, azithromycin used in asthma resulted in no obvious influence on adverse events by a meta‐analysis (Risk ratio 0.89; 95% CI, 0.74‐1.07; *P* = 0.22), which applied in a fixed effects models (heterogeneity *I* ^2^ = 0%, *P* = 0.45) (Figure 7). Moreover, there was no difference in study discontinuation due to adverse effects between the azithromycin and placebo group in the three studies cited above. As Hahn demonstrated,^12^ compared with the placebo, asthma patients taking azithromycin revealed significantly more nausea (n = 10, 29% vs n = 3, 9% for placebo; *P* = 0.016). Johnston SL detected more gastrointestinal and cardiac adverse events in the azithromycin group compared with placebo but with reduced respiratory adverse events (35 vs 24 and 4 vs 2, respectively). Gibson PG reported that azithromycin treatment lead to significantly more diarrhoea (n = 72, 34%) than the placebo (n = 39, 19%; *P* = 0.001).

## 4 DISCUSSION

This systematic meta‐analysis combined the data of 941 asthma patients from seven randomised, double‐blind, placebo‐controlled clinical trials to evaluate the efficacy and safety of azithromycin on asthma. We found that the addition of oral azithromycin to standard asthma care resulted in no statistically significant benefit for lung function (FEV_1_, PEF), airway inflammation or life quality (ACQ, AQLQ). The treatment was mostly well‐tolerated. However, in some studies, there were increases in diarrhoea, nausea, and gastrointestinal and cardiac events as a side effect of treatment.

The protective effect of azithromycin in animal models was demonstrated. Beigelman A et al observed attenuated airway inflammation in an ovalbumin‐induced asthma model of mice after azithromycin treatment, although the mechanism of benefit was unclear.^17^ Liu et al also found that azithromycin significantly reduced the airway inflammation, mucus secretion, airway remodelling, and apoptotic epithelial cells in ovalbumin‐induced asthmatic mice, which might be partially accounted for by maintaining the balance of the Bax/Bcl‐2 ratio and Caspase‐3 level.^18^, ^19^ Findings from the long‐term treatment of chronic asthmatic mice with azithromycin suggest that the alleviated airway inflammation and remodelling might operate through the RP‐39 and MAPK/NF‐κB signal pathways.^20^

Although further mechanistic studies are needed to confirm this, azithromycin has been shown to have immunomodulatory activities by modulating the function of immune cells, such as macrophages, neutrophils, and Th2 cells; all of these cell types participate in the immune response in asthma.^18^, ^21^, ^22^, ^23^ Azithromycin can reduce the activation of pro‐inflammatory transcription factors, including nuclear factor‐κB (NF‐κB) and activator protein 1 (AP1) in lung cells, and subsequently modulate thymic stromal lymphopoietin (TSLP), IL‐6, and IL‐8.^24^, ^25^ The NF‐κB pathway plays an important role in the immune response of asthma. Azithromycin down‐regulated interleukin‐5(IL‐5) production in Th2 cells isolated from asthmatic children and cultured in ex vivo*,* 26 the central role of IL‐5 in the pathogenesis of asthma has been widely demonstrated. Therefore, these findings demonstrate the immunomodulatory properties of the antimicrobial activity of azithromycin and suggest that azithromycin might have beneficial effects in treating asthma. Animal studies and cell culture do not show the complexity and heterogeneity of asthma phenotypes. Thus, cautious analyses are required.

In the included studies, there was more nausea and gastrointestinal and cardiac adverse events in the azithromycin group compared with the placebo. During one study, two patients were withdrawn due to abnormal QTc prolongation,^16^ which is a risk for cardiac arrhythmia and should be seriously considered in the clinical treatment.

Nevertheless, because of the varied heterogeneity of asthma, phenotypes, race, treatment duration, dose, and outcome measures, the effect of azithromycin in clinical trials about asthma patients are inconsistent. Non‐eosinophilic asthma is seemly more responsive to azithromycin therapy than eosinophilic asthma, as Brusselle GG reported.^13^ Add‐on treatment with low dose azithromycin in patients with severe non‐eosinophilic asthma (FeNO lower than the upper limit of normal and blood eosinophilia ≤ 200 ml^−1^) resulted in a significant reduction in the rate of severe exacerbations but showed no beneficial effect when subgroups of eosinophilic and non‐eosinophilic asthma were analysed together. The underlying mechanism is unclear but could be attributed to combined actions of antibiotics and immunomodulation. By contrast, smokers with neutrophilic asthma did not indicate improved symptom control and lung function after azithromycin administration.^14^ We suspect that the short treatment duration (12 weeks) is sufficient to get an effective dosage in this study. In an AMAZES clinical trial conducted in persistent asthma, azithromycin use was associated with reduced asthma exacerbations in both eosinophilic and non‐eosinophilic asthma.^16^ Moreover, these authors did not observe the decreased inflammatory cells in sputum, which was consistent with other studies,^11^, ^14^ indicating that there is no evidence of an antibacterial effect for azithromycin at this dose.

There are several limitations to this meta‐analysis that should be considered. (a) Possible publication bias should not be ignored, where articles with positive conclusions are more likely to be available in the database compared with those showing neutral or negative outcome; (b) Because of the small sample sizes of the included studies, the conclusion of this comprehensive analysis might not be transferable to a large population; (c) Some information was not extracted from the eligible studies, which might influence the interpretation of meta‐analysis; (d) The heterogeneity derived from gender, ethnicity, age, and baseline treatment possibly affected the precision of our conclusions; (e) The dosage of azithromycin and period of conducting the study differed among studies, which might have contributed to the inconsistency; (f) We failed to identify unpublished studies that may alter the outcome; (g) We have not register this study on website for systematic reviews. Although there are several limitations of this study as listed above, we tried to address any study selection bias as described in Methods and we also ensured that the evaluation of each trial was consistently in line with the inclusion or exclusion criteria. Besides, we conducted this study strictly according to The Cochrane Handbook for Systematic Reviews of Interventions 5.1, which could minimised the bias as much as possible.

## 5 CONCLUSION

In conclusion, this systematic review and meta‐analysis has identified available randomised controlled clinical trials and investigated the efficacy and safety of azithromycin treatment for asthma patients. We found no beneficial evidence for azithromycin for asthma patients in lung function, symptom control or asthma exacerbations, and careful consideration should be taken when using azithromycin. Based on our findings, we propose that further prospective cohorts are warranted to assess the effectiveness and adverse events of azithromycin in asthma control.

## CONFLICT OF INTEREST

The authors declare no conflict of interest.
