# Supplementary material for: The efficacy and safety of azithromycin in asthma: A systematic review
Source: J Cell Mol Med. 2019 Jan 19;23(3):1638–46. doi: 10.1111/jcmm.13919 (PMC6378181; doi:10.1111/jcmm.13919)
Supplement: Supplementary file 1 [file JCMM-23-1638-s001.docx]

**Supporting information**

**Table S1: Lung function changes after azithromycin treatment versus placebo**

| Study | Hahn DL  2006 | Piacentini GL 2007 | Hahn DL  2012 | Brusselle GG  2013 | Cameron EJ  2013 | Johnston SL 2016 | Gibson PG  2017 |
| --- | --- | --- | --- | --- | --- | --- | --- |
| FVC | N.M. | N.M. | N.M. | N.M. | N.M. | N.S. | N.M. |
| FEV1 | N.M. | N.S. | N.M. | p=0.686 | p=0.41 | N.S. | N.S. |
| PEF | N.M. | N.M. | N.M. | p=0.378 | p=0.58 | N.S. | N.M. |

N.M., not mentioned; N.S., not significant

**Table S2: Effect of add-on azithromycin treatment on asthma exacerbations**

| Study | Hahn DL  2006 | Piacentini GL 2007 | Hahn DL  2012 | Brusselle GG  2013 | Cameron EJ  2013 | Johnston SL 2016 | Gibson PG  2017 |
| --- | --- | --- | --- | --- | --- | --- | --- |
| Asthma  exacerbations | N.M. | N.M. | N.S. | Rate ratio (95%CI)  1.05(0.63 to 1.76)  p=0.847 | N.M. | N.M. | Hazard ratio (95%CI)  0·65 (0·50–0·85)  p=0·001 |

N.M., not mentioned; N.S., not significant
